# Image-Based Analysis to Dissect Vertical Distribution and Horizontal Asymmetry of Conspecific Root System Interactions in Response to Planting Densities, Nutrients and Root Exudates in *Arabidopsis thaliana*

**DOI:** 10.3390/plants6040046

**Published:** 2017-10-10

**Authors:** Jane Geisler-Lee, Xian Liu, Wei Rang, Jayanthan Raveendiran, Marisa Blake Szubryt, David John Gibson, Matt Geisler, Qiang Cheng

**Affiliations:** 1 Department of Plant Biology, Mailcode 6509, Southern Illinois University Carbondale, Carbondale, IL 62901, USA; marisa.szubryt@siu.edu (M.B.S.); dgibson@plant.siu.edu (D.J.G.); mgeisler@plant.siu.edu (M.G.); 2 Department of Computer Science, Mailcode 4511, Southern Illinois University Carbondale, Carbondale, IL 62901, USA; rangwei1221@gmail.com (W.R.); jayanthan.raveendiran@gmail.com (J.R.); Qiang.Cheng@uky.edu (Q.C.); 3 Program of Environmental Resources & Policy, Mailcode 4637, Southern Illinois University Carbondale, Carbondale, IL 62901, USA; xianliu2014@siu.edu; 4 Department of Computer Science, University of North Carolina at Charlotte, Charlotte, NC 28223, USA; 5 Center for Ecology, Mailcode 6504, Southern Illinois University Carbondale, Carbondale, IL 62901, USA; 6 Institute of Biomedical Informatics & Department of Computer Science, University of Kentucky, Lexington, KY 40506, USA

**Keywords:** *Arabidopsis thaliana*, conspecific root, imaging, root system, skewness and kurtosis

## Abstract

Intraspecific competition is an important plant interaction that has been studied extensively aboveground, but less so belowground, due to the difficulties in accessing the root system experimentally. Recent in vivo and in situ automatic imaging advances help understand root system architecture. In this study, a portable imaging platform and a scalable transplant technique were applied to test intraspecific competition in *Arabidopsis thaliana*. A single green fluorescent protein labeled plant was placed in the center of a grid of different planting densities of neighboring unlabeled plants or empty spaces, into which different treatments were made to the media. The root system of the central plant showed changes in the vertical distribution with increasing neighbor density, becoming more positively kurtotic, and developing an increasing negative skew with time. Horizontal root distribution was initially asymmetric, but became more evenly circular with time, and mean direction was not affected by the presence of adjacent empty spaces as initially hypothesized. To date, this is the first study to analyze the patterns of both vertical and horizontal growth in conspecific root systems. We present a portable imaging platform with simplicity, accessibility, and scalability, to capture the dynamic interactions of plant root systems.

## 1. Introduction

Plants need water, nutrients, light, and space, and compete for these resources in agricultural systems and natural communities [1,2,3,4,5]. This plant–plant interaction is one of the most widely studied [6,7,8,9]. Facilitation occurs mostly in harsh and extreme environments [10,11] while competition commonly occurs in farmlands and moderate environments. But the complication of plant–plant interactions results in different outcomes when subjected to resource limitations [12]. A new understanding of the mechanisms of plant–plant competition is still being developed [13]. 

In intraspecific competition, aboveground tissues of a plant need to grow faster and taller than those of their neighbors in order to shade their competitors [14,15] while belowground tissues expand to exploit soil resources more extensively (than neighbors) by lateral root branching [16,17]. Intraspecific competition studies in the model plant *Arabidopsis thaliana* have been focused on aboveground parts (e.g., shoots) and at the organismal and ecological scale [18,19,20]. In contrast, the mechanisms of these intraspecific interactions at the molecular and genetic scale and on belowground parts (i.e., roots) are poorly understood [21]. The underlying mechanisms have been explored using microarrays in *A. thaliana* [22,23], and more recently, with transcriptome analyses in *Trifolium* spp. [24]. Conspecific shoots of competing *A. thaliana* plants invest in expression of photosynthesis and auxin biosynthesis genes, and sacrifice expression of stress and defense mechanism genes [22]. Conspecific roots invest in expression of nutrient transport genes, especially for the inorganic minerals N, P, and K [23]. 

If representing the fraction of whole plant biomass (M), biomass allocation to aboveground leaves (M_L_) is found to increase with nutrient availability, while biomass allocation to belowground roots (M_R_) decreases with nutrient availability [25]. Nutrients can induce intraspecific recognition/detection [26]. In addition, root systems can secrete root exudates (secondary metabolites) to mediate intraspecific and neighbor interspecific recognition [27]. Recognition of allelochemicals (from root exudates) does not appear to play a role affecting root system growth in avoiding intraspecific competition [28]. Rather, recognition of own roots (from competitors’) is due to physiological coordination among different organs (e.g., stem and leaves) of the same plant [29,30,31,32]. Likewise, aboveground biomass (M_A_ = M_L_ + M_S_, consisting of biomass of leaves and stems) has been modeled to scale equivalently to belowground root biomass (M_R_) [33].

In agriculture, the three-dimension (3D) structure of root systems (coined as root system architecture, RSA) has been taken advantage of in crop improvement [34]. Root systems are plastic, and can be affected by nutrients and the environment [35,36,37]. Root systems can crosstalk between signals from different nutrients in their growing matrix [38,39,40,41]. Sources (e.g., in the proximity or at a distance) and levels (e.g., deficiency or sufficiency) of nutrients can affect physiological co-ordination among different organs in a plant. Interaction of nitrogen nutrients (as “nutritional control”) with primary and lateral roots can mold root systems [42,43,44,45]. The annual plant *Abutilon theophrasti*, for example, was shown to integrate information of nutrient distributions and competing neighbors to optimize root behaviors [46]. Moreover, a “hydropatterning” mechanism—lateral roots produced only on the side of the root in contact with water—was reported; this mechanism could promote initiation and development of lateral roots to grow towards available water [47]. 

The roots and rhizosphere of a plant contain a diverse array of associated microbes, plant hormones, and root exudates [48,49] that can affect biotic interactions and plant productivity [49,50,51]. The *A. thaliana* plant not only contributes root debris as sloughed-off dead root cap cells and live border-like cells to the rhizosphere [52,53,54,55], but the plant roots secrete an array of chemicals as root exudates, promoting a diverse metabolite profile [56,57]. 

Recent automation of high-throughput imaging of 3D objects and development of image processing prompts phenomics to become an important research field. Concurrently, non-destructive imaging in vivo and in situ can advance the understanding of root systems from 2D to 3D, and from early seedlings to mature plants [58,59,60,61,62,63,64,65,66,67,68]. Magnetic resonance imaging (MRI), a medical imaging tool, was modified and applied to study 3D images of roots [69,70,71]. The imaging platform GLO-Roots was developed to allow 3D characterization of root systems growing in soil matrix using transgenic plants with luminescent reporters [72,73]. Among all the open source software available to model root system structures with incorporation of their physiological parameters, the recent OpenSimRoot [74] is the most versatile, which has been refined over the past 20 years [75]. However, these root visualization approaches are complex and logistically challenging to undertake requiring expensive equipment.

Here, we present a portable imaging platform with simplicity, accessibility, and scalability. To assess the effects of planting density and two nutrients and root exudate treatments on conspecific root systems, a media transplant technique was applied, and statistical analyses of root circularity and symmetry were incorporated to unravel growth of conspecific root systems over time. Coupled with the transplant technique and the imaging platform, we present new insights into the interaction of root systems in *A. thaliana*.

## 2. Results

### 2.1. Capturing Root Systems Using a Portable Imaging Platform

To assess conspecific root crowding response to planting densities and media treatments in *Arabidopsis thaliana*, images of the root system of a single (central) plant was captured using a simple, portable and non-destructive imaging platform (see Supplemental Figures S1 and S2 for design). This platform was set up on a lab bench in a small room (in which light could be turned off) and without the aid of a sophisticated automated phenomics facility. A publicly available transgenic line with a green florescent protein marker [76] was used, in addition to non-fluorescent wild type Columbia (Col) seeds (Supplemental Figures S2b and S3b). Three planting densities were tested—five plants per Magenta^R^ GA-7 box (bio-world, Dublin, OH, USA) (density 5, D5) which has been shown in previous studies to have an effect on above ground rosette diameter [22] was compared to three and four plants per box (D3, D4). In addition, two different kinds of treatments were tested to simulate the presence of a plant in the missing positions in D3 and D4 boxes, to see if the treatment had the same effect on growth and root architecture as a live plant. These treatments included plant conditioned media (H), in which a plant was grown in media for 30 days and then removed, and half nutrients (L). These were compared to an undisturbed D5 control (C) and a mechanical procedural control (PC; this was to evaluate how mechanical disturbance of the H and L treatments might affect our results). A modified Canny edge detection algorithm [77] was used to digitize images and depict the root system as pixels in quantifiable data matrices (see Supplemental Methods for further details). An open-source image processing script was developed in MATLAB [78,79], deposited in https://github.com/jbmatthewgeisler/Roots) and deployed on a lab desktop computer. 

The primary roots of *A. thaliana* first grew downward, and then reached the bottom of the Magenta^R^ box at 14 days after planting (DAP) (Supplemental Figure S2b). Next, lateral (secondary) roots emerged, starting from the upper third of the primary root; however, there was no obvious uniformity in the position of lateral roots along the primary root axis between replicates of the same treatment. Each lateral root grew outwards initially, and then downwards, some with additional tertiary roots emerging by 26 DAP, closer to the primary root (for example see Supplemental Figure S3a,b). Conspecific roots avoided close contact, and were at least more than 2 mm apart in all observations. By 26 DAP, still no physical contact of conspecific roots could be observed (Supplemental Figure S3a,b). This observation is in agreement to the findings of self-recognition and avoidance in pea (*Pisum sativum*) roots [80,81,82].

### 2.2. Vertical Distribution of Root Systems

**Figure 1:**
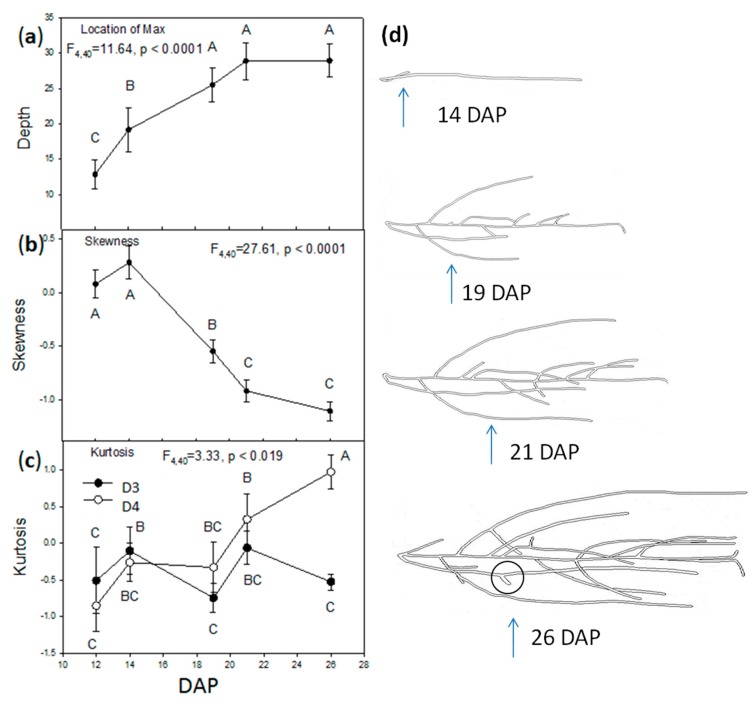
Vertical distribution of root systems through time. Mean (± SE) root pixels along the Y axis from 12 days after planting (DAP) to 26 DAP. (**a**) Location of maximum root density (max) as depth of the root system; (**b**) skewness of density distribution and (**c**) kurtosis. D3 is density of three plants per box while D4 is density of four plants per box. Mean values sharing the same letter are not significantly different (*p* < 0.05). (**d**) Skewness of root density distribution is visible in root skeletons of the same plant (D3, procedural control) over time. There are fewer roots above than below the depth location of maximum roots (arrows) resulting in a negative skew of density at 21 and 26 DAP. Downward growth, spacing of lateral roots, and the appearance of tertiary roots (circled) likely contribute to skewness.

To understand conspecific root system response to planting densities and treatments, the vertical Y axis was first explored. The depth in which the maximum root density occurred was significantly affected by time (DAP) as a main effect, but there were no significant effects of planting density, treatment, or any interactions (Table 1). The total depth of the gel in the Magenta^R^ box was 33 mm. The depth of maximum root density (as “location of max variable”) increased with DAP as roots grew towards the bottom of the Magenta^R^ boxes (Figure 1a; Supplemental Figure S3a). By 19 DAP, roots started reaching the bottom of the box (i.e., depth = 33 mm), and the trend of maximum root density stabilized afterwards. 

Skewness of the vertical root density distribution was affected by separate main effects of planting density and DAP (Table 1). Skewness was significantly more negative for D4 plants (−0.61 ± 0.12) than D3 plants (−0.28 ± 0.10); and while the distribution of roots was initially unskewed at DAP 12 and 14, they became more negatively skewed through time irrespective of treatment or planting density (Figure 1b; Supplemental Figure S4c,f).

**Figure 2:**
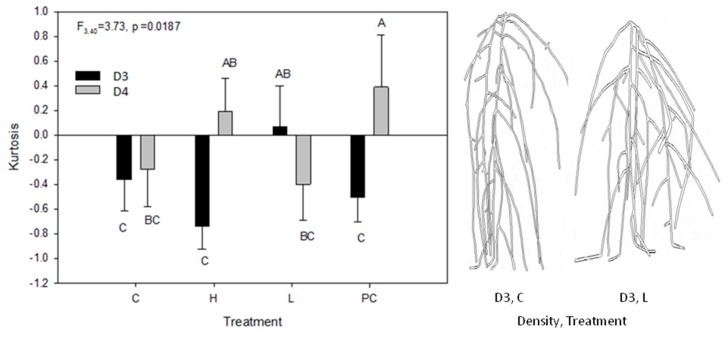
Kurtosis of the root density along the Y axis by planting density and treatment. Mean (± SE) root kurtosis in Y axis view by planting density and treatment. D3 is density of three plants per box, while D4 is density of four plants per box. Mean values sharing the same letter are not significantly different (*p* < 0.05). Treatments C = D5 control, H = plant conditioned media, L = low nutrients, PC = D3/D4 procedural control. Examples of root systems (right) showing platykurtic (flattened peak or negative kurtosis) distribution in D3, H, and a mesokurtic (normal, kurtosis = 0) in D3, L.

Kurtosis of vertical root density was affected by two separate interactions, i.e., between planting density and DAP, and between planting density and treatments (Table 1). From DAP 12 to 21, root distributions had negative or close to zero kurtosis values (Figure 1c; Supplemental Figure S4c). However, D4 plants went from negative kurtosis at 12 DAP (mean k = −0.84) to positive by 26 DAP (k = 0.97), whereas D3 plants remained negative throughout (k = −0.38 ± 0.13). The interaction between planting density and treatment on kurtosis occurred in treatments H and PC roots of D4 plants, which had positive kurtosis compared to negative kurtosis in D3 density plants (Figure 2; Supplemental Figure S4f). There was no significant difference in kurtosis between the two planting densities in C and L treatment plants; mean kurtosis values were negative, or overlapped with zero.

### 2.3. Root Circular Distributions of the Central Plant

**Figure 3:**
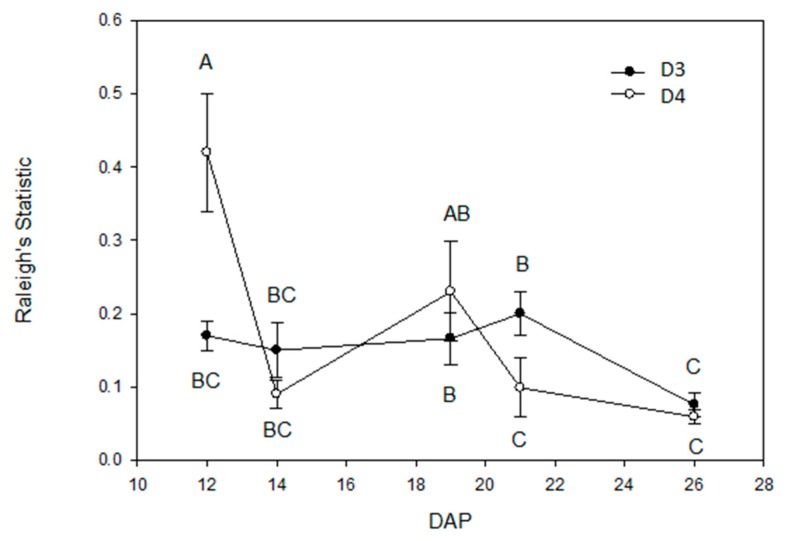
Departure from circular uniformity for root systems in different densities. Circular uniformity by Rayleigh’s statistic was used to show distributions of root systems in combination of X and Z axes. The pixels of the collapsed Y at each coordinate (X, Z) was used to present the central (i.e., position 5) plant’s root systems in D3 and D4 boxes through time from 12 days after planting (DAP) to 29 DAP. Mean values sharing the same letter are not significantly different (*p* < 0.05).

The circular horizontal distribution of roots was calculated by collapsing the vertical dimension into a number on (X, Z) matrices. Root distributions around the stem of the central plants (i.e., position 5 plant) were asymmetric, exhibiting a departure from a uniform circular distribution at all DAPs, in both planting densities and under all treatments (mean Rayleigh’s statistic = 0.17 ± 0.017, mean Watson’s statistic = 371.7 ± 56.4; in both cases *p* < 0.01 @ *n* = 80). Taking the magnitude of the Rayleigh’s statistic as a measure of root asymmetry, there was a significant interaction between planting density and DAP (F4,40 = 2.90, *p* = 0.03), but no effect of treatment, either as a main effect or in an interaction (all tests *p* > 0.05) (qualitatively similar results were obtained from analysis of Watson’s statistic, density F1,40 = 5.81, *p* = 0.02, DAP F4,40 = 4.26, *p* = 0.006, density x DAP interaction F4,40 = 1.91, *p* = 0.13, treatments and treatment interactions *p* > 0.05). Plants in the D4 boxes initially (at 12 DAP) had the highest asymmetry in the distribution of roots around the central plant, which decreased thereafter. By contrast, central plants in the D3 boxes showed no change in asymmetry with time, except at DAP 26, when, in common with plants in the D4 boxes, asymmetry was lowest (Figure 3). 

The (X, Z) matrices were also employed to exhibit growth direction(s) of root systems in windroses (Supplemental Figure S5a,c). Roots from different densities and treatments did not maintain one single direction, instead, they modulated their growth direction(s) between 12 DAP and 26 DAP. For example, the root system from the D3 density and H treatment initially grew toward the interspace between quadrant 3 and 4 (i.e., south) at 12 DAP, followed by moving toward east at 19 DAP and both north and south at 36 DAP (Supplemental Figure S5a,c). When the same (X, Z) matrices were employed with the Y axis number as depth (Supplemental Figure S5a), a 3D heatmap was used to display little growth at 12 DAP, asymmetric growth east at 19 DAP and expansion south at 26 DAP (Supplemental Figure S6a,c).

### 2.4. Aboveground Rosette Size at 28 Days after Planting

**Figure 4:**
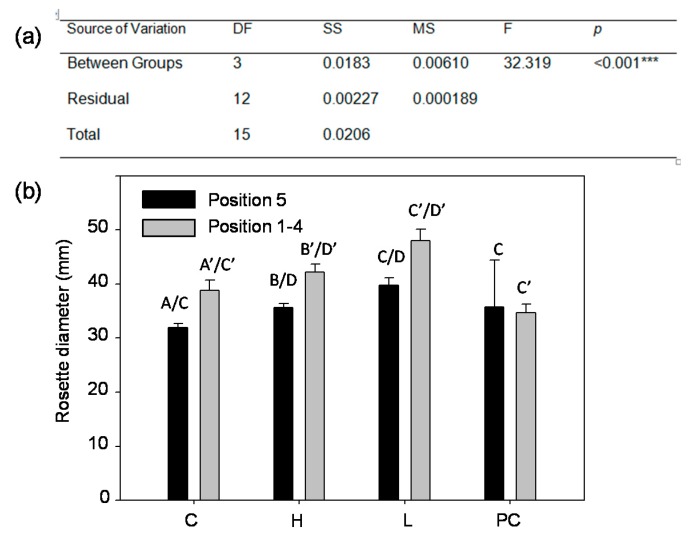
Comparison of rosette diameters in the density 3 plants at 28 DAP. (**a**) Two-way ANOVA tested the variation of treatments (C, H, L, PC) and positions (position 1–4 and position 5) on the rosette diameter at 28 DAP. Position 1–4 were on the four corners of the Magenta^R^ box, while position 5 was central position. Difference can be found in the factors of treatment and position, but there was no significant difference in the interaction of treatment and position. *p* < 0.05. (**b**) There was no difference among all position 5 rosette diameters; but in the position 1–4, H was different from C and PC. Mean values sharing the same letter are not significantly different (*p* < 0.05). C, control; H, conditioned media; L, half nutrients; PC, mechanical procedural control.

At 26 DAP (the last time point to image conspecific root systems in our study), the plants in the Magenta^R^ box were at the Growth Stage S1.12 (i.e., twelve rosette leaves >1 mm in length) according to Boyes and associates [83,84]. At 28 DAP, which is still at the S1.12, the rosette leaves of the density 3 plants were imaged by an overhead camera. Rosette diameters were affected by the two factors, treatment and position, independently without interaction (Figure 4a). Conditioned media (H) previously grew Col plants to 30 DAP (see Section 4), and was hypothesized to contain preexisting root exudates. The rosette diameters in H were different from, and bigger than those in C (control) and PC (mechanical procedural control), but were similar to those in L (half nutrients) (Figure 4b). However, C and PC were not different to each other, nor to L treatment. However, there was no difference in treatments in position 5 root systems.

### 2.5. Diffusion of Inorganic Nitrogen Nutrients through Gel Media and Utilization of the Nutrients at 28 Days after Planting

Diffusion of inorganic nitrogen (as NO_3_^−^/NO_2_^−^ and NH_4_^+^) was tested, to confirm that over a course of 30 days, gel nutrients of the high concentration (as 1/2X MS) and low concentration (as 1/4X MS) could reach an equilibrium, at least in their outermost rings of 3 mm thickness (Supplemental Figure S7a,b). The nutrient level of the initial fresh media (as “Initial” in Supplemental Figure S7c) differed from that of the transplant plugs (as “Transplant” in Supplemental Figure S7d), confirming a nutrient gradient in the H treatment. However, the inorganic nitrogen level did not differ among two planting densities and treatments at 28 DAP (Supplemental Figure S7c).

## 3. Discussion

In contrast to aboveground tissues, the belowground roots are referred to as the hidden half of a plant [85,86]. In this study, we applied a portable imaging platform and a simple media transplant technique (Supplemental Figures S1 and S2) to explore this hidden half. The root systems behaved differently when planting density was altered, media was conditioned by plants or media contained lowered nutrients, although not as hypothesized (Figure 1 and Figure 3). Plants were placed in a grid surrounding a central test plant, which has been shown in previous studies to create a competitive effect on aboveground plant growth and gene expression [22]. Planting density was then lowered by leaving out either 1 or 2 of the surrounding plants in a “missing plant formation”. The remaining plants were hypothesized to detect and grow roots towards the empty space created by the missing plant(s). Treatments were then placed into these missing positions, designed to mimic the presence of a plant in terms of competitive effect, either by the depletion of nutrients or other rhizosphere conditioning by a former plant occupant. *Arabidopsis thaliana* also shows ontological changes when presented with conspecific competitors [18,19,22,87,88,89]. The efficiency of root lateral branching is hypothesized to minimize energy expenditure (i.e., growth) in harvesting of nutrients, and new roots are thus expected to avoid low nutrient or plant occupied spaces. In the absence of competitors, this response comes from directing root growth along a concentration gradient of increasing nutrients [90]. Thus, changes to the root system were expected, but the observed changes were more subtle than simply extending asymmetrically into the unoccupied space. 

A significant change was observed in the vertical (Y axis) density distribution of roots in response to planting density (number of competitors) and time (days after planting, DAP). Root systems grew with a negative kurtosis, meaning an evenly vertically distributed root system at early time points in all plants. At later time points, the lower planting density (D3) remained negatively kurtotic in contrast to a shift to positive kurtosis (where more roots occurred at a specific depth) in higher planting densities (D4) (Figure 1c). A negative shift in the skewness of the vertical distribution of the root system was also observed in all plants with time. In this study, obvious changes in horizontal (X, Z) root distribution by planting density or treatment were not observed. Although root systems were indeed shown to be asymmetric (departed from a circular distribution), a consistent mean direction in root growth was not maintained over time. The asymmetry of the root system as measured by the Rayleigh’s statistic decreased with DAP (Figure 3), meaning as the root system “filled in” over time, it approached a more circular distribution. This effect may come from periodic spiral-like lateral root branching along the primary or main root that is regulated by oscillating gene expression of some transcriptional regulators in *A. thaliana* [91]. Primary roots originate from the embryo, while lateral roots are initiated and formed in the pericycle, a thin cell layer of plant tissue between the endodermis and the phloem (of the vascular bundle). Initiation of lateral root primordia is regulated by auxin [92] and endodermal feedback (due to its volume loss) [93,94]. Laskowski and ten Tusscher suggested three mechanisms for periodic lateral root initiation [95]. This periodicity of lateral root formation could be affected by external abiotic (e.g., nutrients and root exudates) and biotic (e.g., planting densities) factors/stimuli, and thus, overall root system structure/architecture can be changed. In this study however, the roots of the central plant did not seek out the empty spaces in the media, and grow asymmetrically towards them.

An important product of this study was the proof of the method itself. A simple, low cost system is presented to non-destructively explore and quantify the belowground root system over time. 3D heatmaps (Supplemental Figure S5) and 2D windroses (Supplemental Figure S6) complementarily depict growth and direction(s) of a root system. This method also easily quantifies vertical phenotypic characteristics (Y axis) of root systems, such as kurtosis and skewness, and shows changes during the development of the root system. The optically clear gel can be manipulated to apply or remove nutrients in different segments of the media, either horizontally, as in the L and H treatments of this study, or vertically, creating layers. In addition, any transparent container (e.g., larger than Magenta^R^ boxes) can be utilized to scale up the experiments. Square plastic boxes were chosen to avoid the optical distortion of a curved container, and only four images (one from each face) were used in this study. The root systems of *A. thaliana*, when grown in agar media at the laboratory, were reported to be regulated by the uptake of externally provided sucrose (in the media) in their aerial leaves [96]. The root systems of *A. thaliana* are also controlled by loci and environments [97,98]. In this study, the media were rich with inorganic nitrogen (Supplemental Figure S7c,d) and contained sucrose (see Section 4); roots of planting densities and treatments were observed to explore the lower gel segment. Thus, root vertical distribution skewness went from positive (i.e., growing more in the upper segment of the media) to negative (more in the lower segment of the media) over time (Figure 1b; Supplemental Figure S4c,f). In a nutrient-localized study in barley (*Hordeum vulgare* cv. Proctor), seminal (adventitious lateral) root systems grew only in the nitrogen-rich segment of soil matrix, but not in the nitrogen-deficient segment [99]. The same phenomenon of nutrients corresponding to lateral root growth also applied to phosphate, but not to potassium [99]. This phenomenon did not apply to our study because of its homogeneous nitrogen source in the media (see Section 4). 

In addition to nutrients, root systems can be affected by associated environmental factors; available water shapes hydropatterning [47], while strigolactones affect lateral root branching [100,101,102,103]. The rhizosphere with root exudates, sloughed off root cap cells, and rhizosphere microbial community can influence the structure of root systems [104]. The complexity of microbiomes [49,50] will affect how solutes move in the rhizosphere [105,106]. All these factors could be manipulated through our simple transplant technique, and tested in hypotheses. Manipulation of growth media with precise ingredient(s) and concentration(s), gradients included, is feasible. As diffusion of inorganic nitrogen occurred in our media (Supplemental Figure S7a,b), studies on nutrient gradients are possible. Although the half strength media (as L; containing ¼ strength of MS media) did not seem to effect in this study (Supplemental Figure S6), more stringent concentrations, as much lower concentrations than ¼ strength of MS media, could also be tested.

Physiological coordination has been observed between shoot and root development in recent studies. Shoot branching is found to be controlled by strigolactone synthesized in the roots [107]. Shoot development and root development of *A. thaliana* are under mutual signaling according to its available resources. Puig and associates provided a first and direct link of the NITRATE TRANSPORTER 1.1 (NRT1.1) transporting auxin [108]. That link shows the tight knitted coordination between auxin (produced in the stem), root development, and nitrate availability in soil. Conversely, it is physiological coordination of aboveground tissues (leaves and stems) of the same plant that is responsible for avoiding conspecific root systems in the proximity [29,30,31,32]. In our study, the vertical kurtosis between the root systems of the conditioned media (H, with root exudates) and ¼ strength of MS media (L) differed; H was negative while L was positive (Figure 2). However, the biometrics of their aboveground rosette diameters did not differ (Figure 4). 

## 4. Materials and Methods 

### 4.1. Seeds, Seed Germination, Conditioned Media Preparation, and Treatment Setup

*Arabidopsis thaliana* ecotype Columbia wild type (Lehle Seeds accession Col-0; herein referred to as Col) and a transgenic GFP marker line (herein referred to as ER::GFP), also in Col-0 background with a green fluorescent fusion protein that was localized in vivo to the endoplasmic reticulum (ER) [76], were used to isolate from the root systems of a single plant amongst neighbors in fluorescence imaging. Seeds were sterilized with 15% Clorox for 15 min, and washed thoroughly with sterile distilled water 3 times. The seeds were sown on the surface of 150 mL gel growth media. The media contained 1/2 strength of MS medium (Cat # 30630058-4, plant media.com) plus 1% sucrose, pH adjusted to 5.7 and with 0.3% (*w*/*v*) gellan gum (Prod # G434, PhytoTechnology LaboratoriesR) for support in a Magenta^R^ box (GA-7, dimensions = 76.2 mm × 76.2 mm × 101.6 mm; bio-world, Dublin, OH, USA). Half strength of MS medium is non-limiting for the growth of *A. thaliana*. Magenta^R^ boxes were chosen due to their semi-transparency and square growth area. Gellan gum is approximately 80% optical transparent, and suitable for high resolution digital imaging [109]. Col and ER::GFP seeds were used to help differentiate root distribution in growth media. No visible differences in root or shoot growth were observed in the ER::GFP plants when compared to Col plants in this medium or on soil. 

Seeds were sown in five designated positions, one central (position 5) and four adjacent to each corner (position 1–4) using a plastic sheet (Staples^®^ Write-On Transparency Film, Staples, Carbondale, IL, USA) for exact positioning according to Supplemental Figure S1, with one seed per position. After 4 days of 4 °C cold and dark stratification, the boxes were transferred to a Percival AR-66L2 growth chamber at 20 °C with a light cycle of 16 h (i.e., long days) and a light intensity of 250 μmol s^−1^ m^−2^. The clear Magenta^R^ boxes sat in black pots to prevent root exposure to light and production of reactive oxygen species [110]. To create conditioned medium, additional boxes were grown to 30 days after planting (DAP), and the position 5 (center) plant was removed, and its gel plug was harvested by a #7 hole puncher with an internal diameter of 2.54 cm. The gel plug was immediately used as “conditioned media” in the experiment. Thus, the conditioned media would potentially contain root exudates, and other conditioning of the rhizosphere done by the former occupant plant.

In the main experimental series, ER::GFP seeds were sown in the central position (as position 5) with either Col plants or media (control, conditioned, or nutrient reduced) in the other four positions. Positions were numbered 1–4, starting at the left on a marked side (A) and counting clockwise around the box (see Supplemental Figure S1c and S2 for exact layout). The substitution of a plant with media resulted in three densities—density of five (D5 all positions occupied with a plant) as a control, and a density of four (D4, four plants per box, one position substituted) and density of three (D3, three per plants per box, two positions substituted). Thus, a single position (position 3) was missing a Col plant in the D4 boxes, while two positions in opposite corners (position 1 and position 3) were missing Col plants in the D3 boxes. The position(s) missing Col plant(s) were replaced with media with different treatments. At both D3 and D4 densities, three treatments were tested: a fresh media with 50% nutrient concentration (i.e., ¼ strength MS) coded L for low nutrient; a “conditioned media” as described above (coded as H for high root exudates); and a mechanical procedural control (coded PC) with the gel medium uplifted and returned immediately to simulate any mechanical stress or structural alteration of the gel from the media transplant (Supplemental Figure S1c). The media transplant technique was performed by applying a #7 hole puncher to drill a gel stick from either previously grown gel (for H) or reduced half nutrients (for L) (refer to Supplemental Figure S1b for hole drilling and gel plug replacement). 

### 4.2. Fluorescence Image Acquisition and Processing

Images of roots were taken from four sides of each Magenta^R^ box (A–D, clockwise), such that two sides (A, C) corresponded to the X-axis as horizontal and Y-axis as vertical, and another two (B, D) corresponded to the Z-axis as horizontal and Y-axis as vertical. Supplemental Figure S2 shows the setup for camera and illumination. To image only the center ER:GFP plant, fluorescence photography using a Nikon D5000 12.3 MP DX digital single-lens reflex camera covered with a 505 nm long pass emission filter. Illumination was with a 3W CREE XP-E blue LED lamp (465–485 nm) with a 485 nm short pass excitation filter. Each box was marked with a label for treatment and replicate numbers and for orientation (side A was labeled, others proceeded clockwise). Images were taken on four sides A–D, clockwise (refer to Supplemental Figure S2). Images were taken with an F-stop 8, to increase focal depth. Root images were taken at 12, 14, 19, 21, and 26 days after planting (DAP). At 28 DAP, images were taken of the shoots from above for rosette diameter measurements, after the lids of the boxes were removed. At 28 DAP, the plants were still in the rosette development stage, also known as principal growth stage 1 according to Boyes and associates [83,84]. 

Root images were processed in Adobe Photoshop to help isolate the roots of the (green) ER:GFP plants growing in position 5 from the gel media, conspecific (non-fluorescent) competitors, and other artifacts (i.e., labels and box reflection). Images were also taken with white light for comparison. The processed images were cropped to a standard size without losing any roots (Supplemental Figure S3), and were then processed using the Canny detection program in MATLAB [77,78,111]. The resulting images are termed “root skeletons” (Supplemental Figure S3d). A few more non-root pixels (artifacts) present were removed manually in Adobe Photoshop (see the black dots in Supplemental Figure S3c), and portions of the root not captured were added/connected manually as well (Supplemental Figure S3d). Root skeletons were processed further using custom programs in MATLAB in Dell Precision server computer with 4 Xeon processors and 64 Gb RAM memory running Linux, to generate quantified root density per-pixel in matrices for statistical analysis (scripts of these programs were deposited in GitHub, https://github.com/jbmatthewgeisler/Roots). A total of 20 plant boxes were examined at 5 time points (2 densities * 4 treatments * 2 replicates) for a total of 320 images (16 boxes * 5 time points * 4 sides of each Magenta^R^ box = 320). The entire procedure, from a cropped image of the root to quantified root density matrices, are detailed in the Supplemental Methods.

### 4.3. Analysis of Inorganic Nitrogen Contents in the Gel Media and their Diffusion

Inorganic nitrogen (nitrate/nitrite and ammonia) analysis of the replacement gel blocks (for H treatments) was made at 30 DAP, when the preparatory setups were established. The same analysis of the gel media for the position 5 plants was made at 28 DAP, after the experimental study was completed. The extraction method for gel media followed that of Keeney and Nelson [112]. A plug of gel media was drilled using a #7 hole puncher, and 10 g of gel was weighed into a 125 mL Erlenmeyer flask. Then 50 mL 2 N KCl was added, and shaken at 200 rpm for one hour. Afterwards, the mixture was filtered through a 0.4 μm filter. The filtrate then was segmented by flow analysis at an OI Analytical Flow Solution IV analyzer (OI Analytical Corp., College Station, TX, USA) to quantify nitrate (NO_3_^−^) and nitrite (NO_2_^−^) levels. The nitrate test followed USEPA method 353.2 [113], in which nitrate is first reduced to nitrite by cadmium metal.

Diffusion of inorganic nitrogen in gel media over 30 days was assessed in the following experiment. Three plugs (drilled by a #7 hole puncher) of half strength MS nutrient media (i.e., C treatment) were inserted into quarter strength nutrient media (i.e., gel plugs in the L treatment) in Magenta^R^ boxes, where they remained for a course of 30 days in the aforementioned growth chamber. Outer and inner 3 mm thick rings of gel were scrapped/removed from the half strength gel plugs and quarter strength surrounding media, respectively, for inorganic nitrogen contents [112,113].

### 4.4. Statistical Analysis

An XZ root matrix was generated by taking average pixel counts from the XY and ZY images of each plant, reducing each column (collapsing the Y-axis) to an estimated number of root pixels at that XZ coordinate. The matrix was transformed to a table of polar coordinates in R [114] by choosing a center position (the XZ position of the hypocotyl on the gel image). A heatmap and windrose was generated as if viewing the root system from overhead, but capturing the total root quantity at each XZ coordinate. Complete procedural details and background theory were presented in Supplemental Methods. Repeated measures mixed model analysis was used to test the effects of total planting density (3 or 4 plants per box), treatment (C, H, L, PC) and their interaction (density x treatment) through 26 DAP on root pixel counts from the top to the bottom of the box (i.e., vertical Y axis view) and in the overhead (plane X–Z axis view). Root counts were expressed as location of maximum depth, skewness, and kurtosis in the Y axis, and as Rayleigh’s and Watson’s measures of departure from a uniform circular distribution, and skewness and kurtosis in the X–Z axis views, calculated in R “circular” package [114] following Pewsey et al. [115]. Log transformed values of the location of maximum depth and Rayleigh’s and Watsons’ statistics were used in the analysis to improve normality. The repeated measures mixed model analysis was conducted in SAS Ver 9.4 using the Kenward–Roger correction to estimate degrees of freedom [116]. Post hoc means separation tests were conducted on least square means. One-way ANOVA in SigmaPlot Ver 11.0 was used to determine whether inorganic nitrogen contents in the different treatments were different. *p* < 0.05.

## 5. Conclusions

Our proof-of-concept study exhibited that our easy-to-operate and portable imaging platform could capture both phenotypic characteristics of root systems in 3D—combination of 2D of X and Z axes and vertical Y axis. This study also highlighted the potential and advantages of the simple transplant technique, and paved a scalable framework with precise manipulation of added ingredients for future studies in ecological questions (e.g., intraspecific competition) and possible environmental problems (e.g., ecotoxicology).

## Figures and Tables

**Table 1:** Characteristics of root systems along the Y axis over time.

Effect	DF	Location of Max (F)	Skewness (F)	Kurtosis (F)
Density	1	0.34	10.09 **	4.21 *
DAP	4	11.64 ***	27.61 ***	4.09 **
Density * DAP	4	0.71	0.26	3.33 *
Treatment	3	1.99	1.30	0.45
Density * Treatment	3	0.21	0.66	3.73 *
DAP * Treatment	1	0.78	0.73	1.18
DAP * Density * Treatment	12	0.80	1.17	1.80

F-statistics from repeated measures mixed model analyses testing the effects of DAP, density, treatment, and interactions on location of maximum pixels, skewness (measure of symmetry), and kurtosis (measure of tailing) of root systems in the Y-axis view. *p* < 0.05 *, *p* < 0.01 **, *p* < 0.001 ***. DF, degrees of freedom; F, F value.

## Supplementary Materials

The following are available online at www.mdpi.com/2223-7747/6/4/46/s1, Figure S1: Experimental setups with two densities and four treatments, Figure S2: Setup of fluorescence imaging, Figure S3: Image processing, Figure S4: Histograms of two roots in the D3 density at 26 DAP, Figure S5: 3D heatmaps of root horizontal distribution, Figure S6: 2D root density windroses, Figure S7: Inorganic nitrogen nutrients of diffusion assays and effects of densities and treatments, Method S1: Root recognition and image digitalization, Method S2: Assignment of polar coordinates and windrose plots.
