# Supplementary material for: Image-Based Analysis to Dissect Vertical Distribution and Horizontal Asymmetry of Conspecific Root System Interactions in Response to Planting Densities, Nutrients and Root Exudates in Arabidopsis thaliana
Source: Plants (Basel). 2017 Oct 10;6(4):46. doi: 10.3390/plants6040046 (PMC5750622; doi:10.3390/plants6040046)
Supplement: Supplementary file 1 [file plants-06-00046-s001.pdf]

Supporting Materials: Image-Based Analysis to Dissect Vertical Distribution and Horizontal Asymmetry of Conspecific Root System Interactions in Response to Planting Densities, Nutrients and Root Exudates in *Arabidopsis thaliana*

Jane Geisler-Lee, Xian Liu, Wei Rang, Jayanthan Raveendiran, Marisa Blake Szubryt, David John Gibson, Matt Geisler and Qiang Cheng

1. Supporting Information

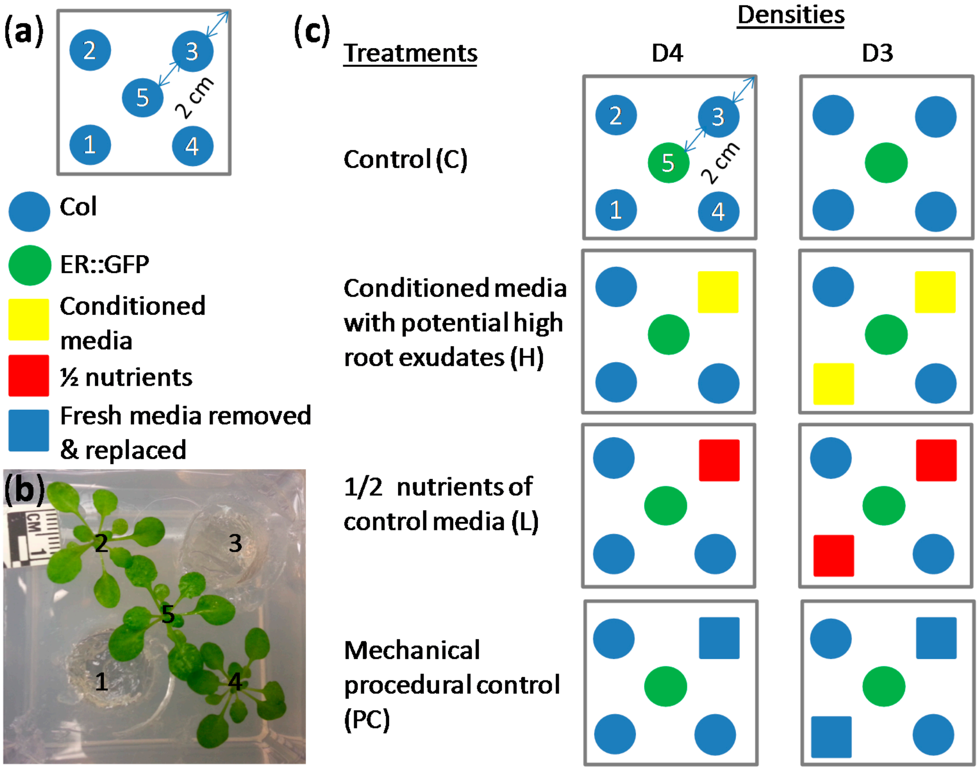

Figure S1. Experimental setups with two densities and four treatments. (**a**) Positions 1–5 and the distances of 2 cm between corner position(s) (1–4) and central position (5) were illustrated. Position 1-4 started at the lower left and clockwise. Circles were Columbia (Col, blue) and ER::GFP (green), while squares indicated conditioned media (yellow), half nutrients (i.e., 1/4X of MS media) (red) and fresh media removed and replaced (blue). (**b**) A representative image of the H treatment in the D3 density at 26 DAP. (**c**) The control setup C was five plants per box with four Col seeds (blue circles) on the position 1–4 and one ER::GFP (blue circle) at the position 5. Two densities were set up, five seeds (as density 5, D5, as control) vs. four seeds (D4) per box and five seeds (D5, as control) vs. three seeds per box (D3). In the D4 density, the medium of the position 3 was replaced with the conditioned medium, with half nutrients, and with fresh media removed and replaced as mechanical procedural control. In the D3 density, the media of the two positions, 1 and 3, were modified.

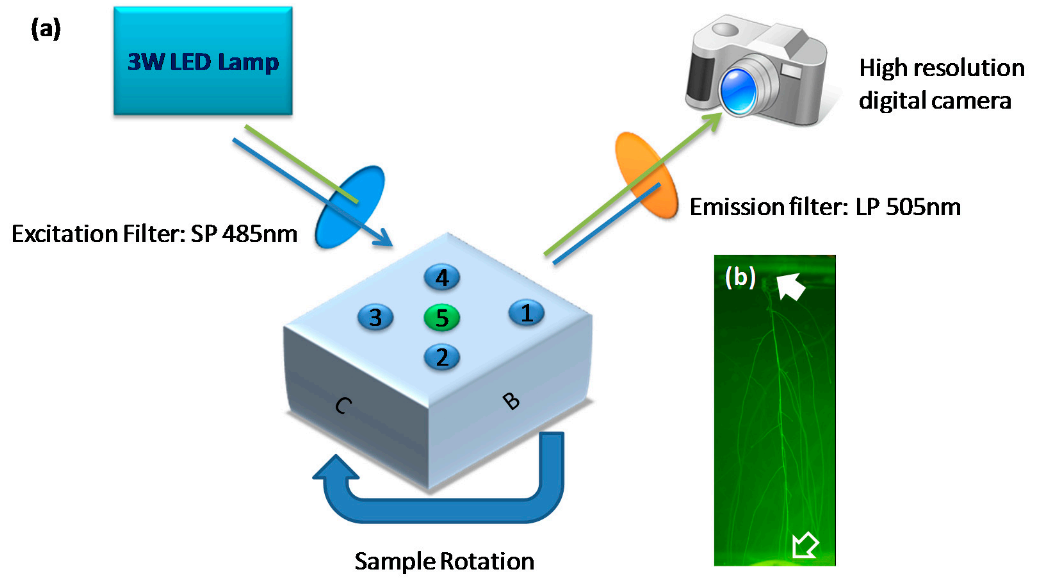

Figure S2. Setup of fluorescence imaging. (**a**) Illumination and image-taking by a camera were placed as perpendicular to each other. An emission filter of long path (LP) 505 nm was used to block the excitation wavelengths (505 nm) and permit only green emission wavelengths to pass toward the camera; an excitation filter of short path (SP) 485 nm was used to permit only blue light to pass through on the way toward the roots in a Magenta^{R} box. Arabidopsis Columbia ecotype wild type seeds (light blue circles) and ER::GFP seeds (green circles) were sown according to the setups in Figure S1. Images were taken from side A, then side B, side C and finally side D. (**b**) A representation of an ER::GFP root system. White solid arrow points to the junction of the root and stem and surface of the gel; hollow white arrow points to the bottom of the box when the root reached it.

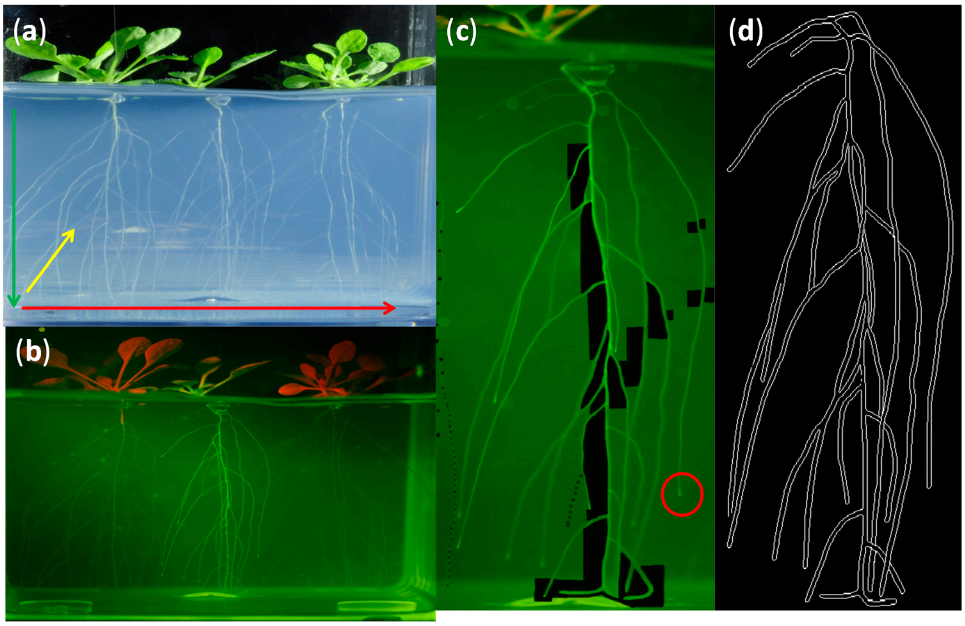

Figure S3. Image processing. (**a**) Raw image of root systems taken from white light; (**b**) the same image taken from blue light; (**c**) after Adobe Photoshop processing of the image to acquire the image of the position 5 (central) root; (**d**) Post-Canny editing to acquire the "root skeleton". The image was from side B (refer to Figure S2) of the Control box (refer to Figure S1) in the D3 density at 26 DAP. The red circle highlighted a root tip. In (**a**), red arrow presented X1 axis, yellow Z1 axis and green Y axis.

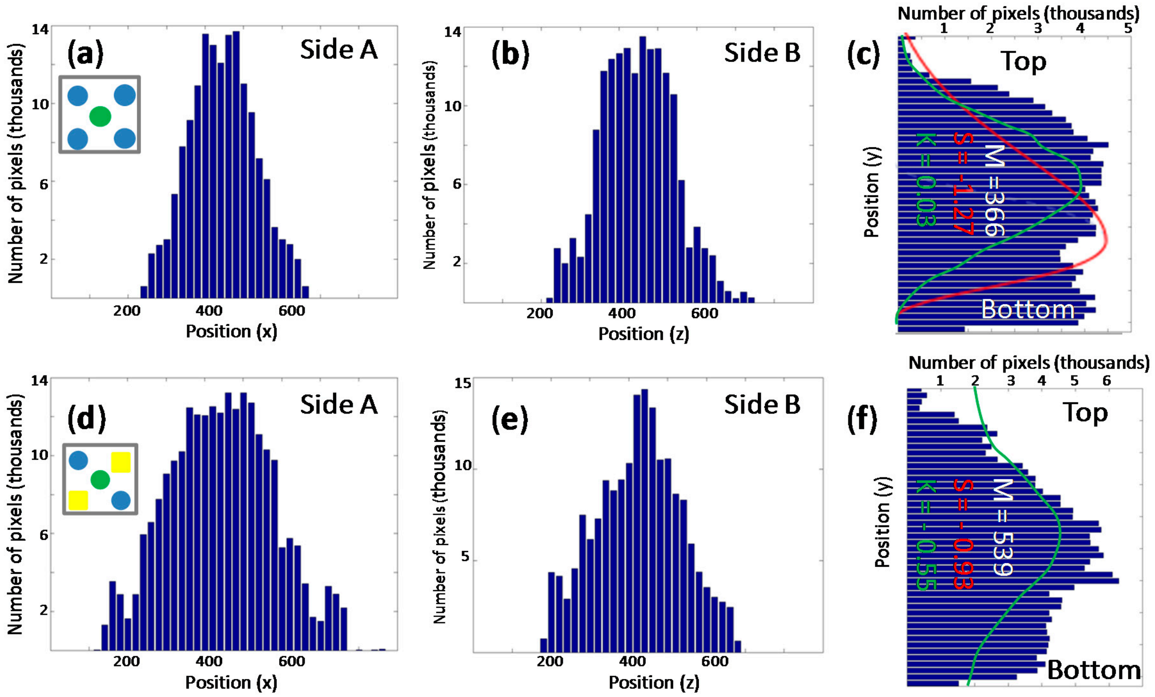

Figure S4. Histograms of two roots in the D3 density at 26 DAP. (**a–c**) came from a "C" treatment while (**d–f**) from an "H" treatment. (**a**,**d**) are pixel distribution of the root from Side A (refer to Figure S2; i.e., X_1 axis); (**b**,**e**) from Side B (Z_1 axis); (**c**,**f**) from top to bottom as depth of a root (Y axis). Negative skewness (S in Red) occurs in (**c**,**f**) but negative kurtosis (K in green) only in (**f**), not (**c**). M is modal depth (in white).

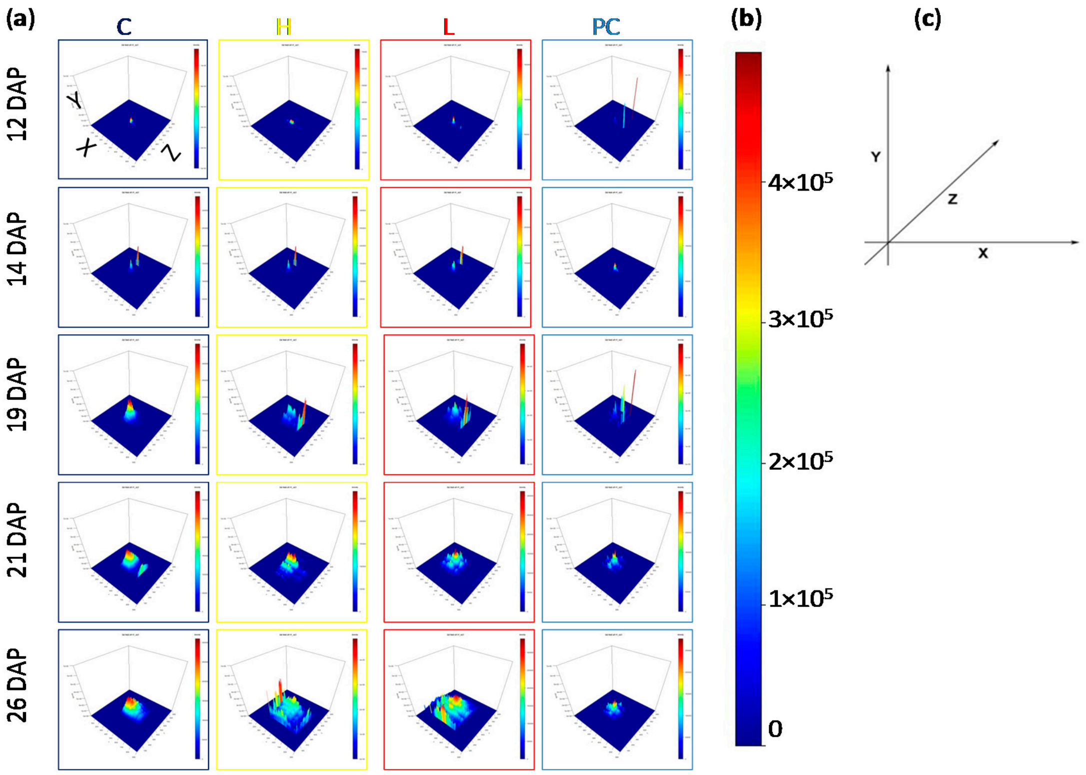

Figure S5. 3D heatmaps of root horizontal distribution. (**a**) 3D vertical representatives of four treatments (C, H, L, PC) of the D3 (density 3 plants per box), replicate 1 from 12 days after planting (DAP) to 26 DAP. (**b**) Pixel intensity from 0 (dark blue) to more than 4×10^5 (dark red). (**c**) A representation diagram of X, Z and Y axes.

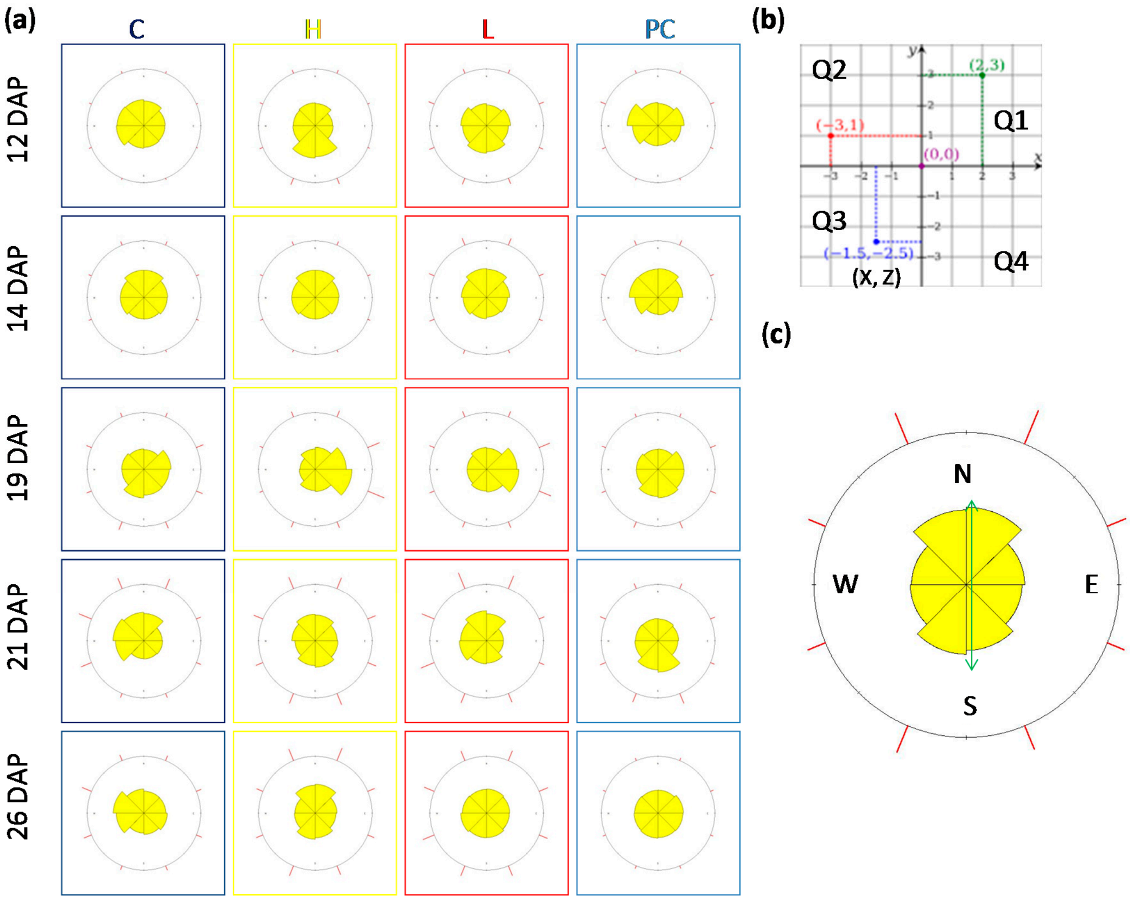

Figure S6. 2D root density windroses. (**a**) Based on Cartesian Coordinates (X, Z) converted to polar coordinates, windroses of 8 wedges (45 degrees per wedge) were made to illustrate the growth direction(s) of root systems. The data are of the same in Figure S5A, four treatments (C, H, L, PC) of the D3 (density 3 plants per box), replicate 1 from 12 days after planting (DAP) to 26 DAP. (**b**) A representative of Cartesian Coordinates with four quadrants (Q1–Q4). (**c**) A windrose representative from the H root system at 26 DAP. Each red tick presents the mean pixels in that wedge. The green double arrow pointed the root system growing toward both south (S) and north (N).

(a)

Source of Variation	DF	SS	MS	F	*p*
Between Groups	3	0.0183	0.00610	32.319	<0.001***
Residual	12	0.00227	0.000189		
Total	15	0.0206			

(b)

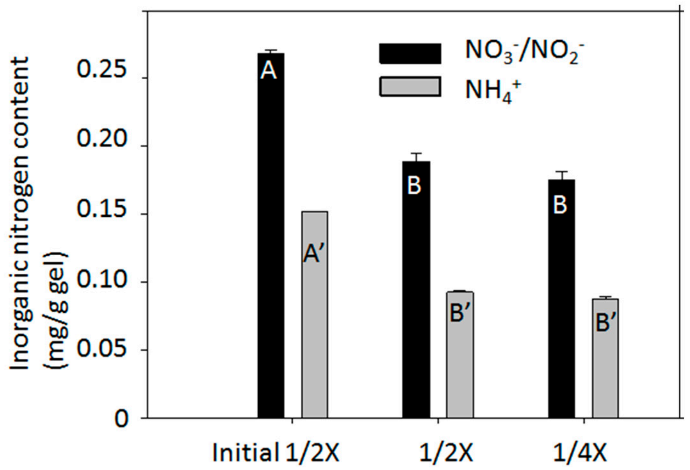

(c)

Source of Variation	DF	SS	MS	F	*p*
Between Groups	8	0.00790	0.000987	5.711	0.004**
Residual	12	0.00207	0.000174		
Total	20	0.00997	38.373		

(d)

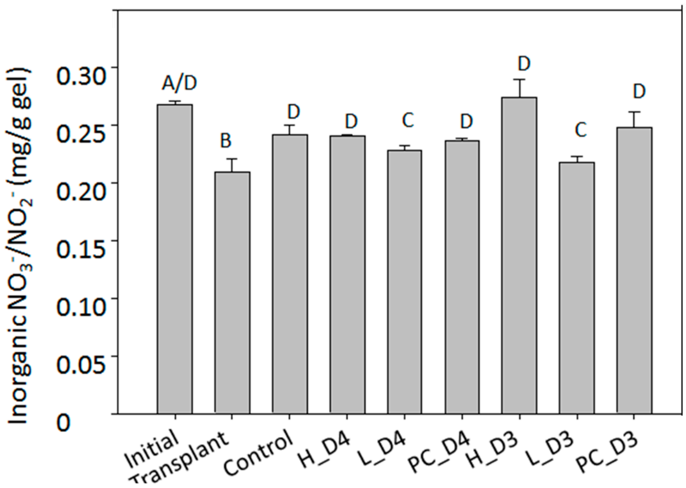

Figure S7. Inorganic nitrogen nutrients of diffusion assays and effects of densities and treatments. (**a**) Diffusion assays were done by having the plugs of initial fresh 1/2 strength of MS media (as "Initial 1/2X") inserted in the 1/4 strength of MS media (1/4X) over a course of 30 days. After 30 days, the inorganic nitrogen content in the plugs (as "1/2X"), at least 3 mm thickness of its outer ring, was similar to that in its surrounding (as "1/4X"). One-way ANOVA showed differences between three (Initial 1/2X; 1/2X; 1/4X). (**b**) Contents of inorganic NO_2^-/NO_3^- and NH_4^+ in Initial 1/2X differed from those in 1/2X and 1/4X. (**c**) Effects of densities (3 plants or 4 plants per box as D3 or D4) and treatments high (H) with root exudates, low (L) nutrients (i.e., 1/4X MS media), and mechanical procedural control (PC), on the changes of inorganic nitrogen nutrients in the position 5 (i.e., central position; refer to Supplemental Fig. S1 and Supplemental Fig. S2) after growing Arabidopsis for 28 days. One-way ANOVA using of the initial fresh media (as "Initial") as control showed difference between groups. (**d**) The content of the Initial was different from that of the transplant tube inserts (as "Transplant"), four plants (per box) in 1/4X MS (L_D4) and three plants (per box) in 1/4X of MS (L_D3). Mean values sharing the same letter are not significantly different ($p < 0.05$).

2. Supplemental Methods

2.1. Root Recognition and Image Digitalization

Pattern recognition techniques common in medical imaging were used to extract salient features from the digital images of roots [1,2]. However, due to the existence of artifacts in the root images caused by lighting reflections by the Magenta[R] box, image processing techniques were also applied to remove the artifacts and to retrieve the root image proper. Then we used an automated region of interest (ROI) detection method, the so-called aROIs method (i.e., using an algorithm to detect ROIs), to find a rectangular region that contains the roots for each image [3]. Since Arabidopsis roots were the main objects in our images, human defined ROIs (i.e., hROIs) can be also used. This operation removed the container edges from the images while retaining the roots only. Afterwards, a series of image processing techniques were applied to extract the root architecture from the images, including improving edge detection with morphological operations to connect the gaps in the roots resulted from edge detection while removing noise.

Canny edge detection algorithm [4] was combined with morphological operations that was capable of detecting line segments [5,6]. Canny edge detector achieved three basic objectives. First, it has a low error rate. All true edges should be found and there should be no false edges. Second, it localizes the edges well. The edge found by the detector should have the minimal distance from the center of the true edge. Third, it produces a single edge point response. No multiple edge points are identified where only one edge point exists. To achieve these objectives, the algorithm consisted of the following four stages. Stage 1. Noise reduction by smoothing the original image with a Gaussian filter *G(x, y)*,

$$G(x,y) = \exp\left(-\frac{x^2 + y^2}{2\,\sigma^2}\right), \tag{1}$$

where σ is a parameter representing the window size. In this study, we used σ = 1.4 to create a 5 x 5 low-pass filter to spatially convolve with the original image *I(x, y)*,

$$I_s(x,y) = G(x,y) * I(x,y), \tag{2}$$

where * represents spatial convolution. Stage 2. Computation of the gradient magnitude and angle of the images. To find the directions of the edges, the horizontal, vertical and diagonal edge detectors such as Roberts, Prewitt, and Sobel filters [5] were first applied to the blurred image $I_s(x,y)$. Then the magnitude and angle were calculated as

$$M(x,y) = \sqrt{g_x^2 + g_y^2}\,, \theta(x,y) = \tan^{-1}\frac{g_x}{g_y}, \tag{3}$$

Stage 3. Suppression of the non-maxima. The angles were rounded to four angles, for example, -45, 0, 45, and 90 degrees. The magnitudes *M(x, y)* often contained wide ridges, which were examined to determine whether or not they were local maxima [4]. As a result of this operation, thin edges $g_N(x,y)$ which were either 0 or a local maximum, could be obtained. Stage 4. Detection and linking of edges through hysteresis thresholding which required double thresholds T_l and T_h with their ratio being two or three to one. Two auxiliary images g_{NL} and g_{NH} were created where g_{NL} (g_{NH} respectively) contained all pixels satisfying $g_{NL} \geq T_l$ ($g_{NH} \geq T_h$ respectively). Then all nonzero pixels were eliminated from g_{NL} by letting

$$g_{NL}(x,y) = g_{NL}(x,y) - g_{NH}(x,y), \tag{4}$$

Subsequently, simple connectivity analysis considering 8-neighbor connectivity was performed to link the edges for obtaining longer edges.

After using the Canny edge detector, it was noticed that some faint parts of the roots still became gaps (Figure 3D). The most parts of the edges were extracted; however, they could still be

disconnected because of the lighting variations, for example, caused by reflections. We adopted morphological operations, including dilation followed by erosion, or thickening after thinning, to further process the image so that discontinuous parts of the edges (of roots) could be connected and the root of one plant can be traced automatically. After a series of such processing, the resultant images were used to extract salient features for pattern recognition. For example, histograms of the root images were constructed, based on which the statistical distributions of the roots were obtained for modeling growth patterns. More specifically, histograms of the roots following horizontal scanning lines or vertically scanning lines were built for analysis. The histograms were then depicted into pixels for downstream statistical analysis. Supplemental Fig. S3D showed the extracted roots of Figure 3C after the above-described sequence of image processing steps. Supplemental Fig. S4A and 4B shows the distribution of roots in one dimension (axis X) and presented axis X of root density from both Side A and Side C, respectively; Supplemental Fig. S4C and 4D in the other dimension (axis Z) and presented axis Z of root density from both Side B and Side D, respectively. Next, two dimensions (i.e., axes X and Z) of root density were reconstructed for the position 5 roots (for all the groups and treatments) by collapsing Y onto an XZ matrix from a three dimension (axes X, Y, Z) data collection. The XZ matrix contained numbers representing the sum of root pixels in each Y-column at that XZ coordinate. Relative total root mass (using the assumption that roots in each pixel were approximately equal in mass density) was calculated by collapsing X, Y and Z into a single number representing the sum of all root pixels for each plant.

2.2. Assignment of Polar Coordinates and Windrose Plots

The rows and columns (m, n) for the X-axis and Z-axis data matrix of root distribution for each plant was converted to generate (x, z) coordinates centered on the plant. The center (0, 0) was found by locating the point in the plant where the hypocotyl and root junction originating from the seed sat at the surface of the media in each images taken of X and Z sides. The exact distance from the edge was determined using Image J [7]. Often this resulted in a "best guess" scenario that was very close to the true center. The converted matrix was loaded into the R environment [8] and run by the IDLE's (an integrated development environment for Python) graphical user interface (GUI) for Python. The Cartesian coordinates (x, z) for each matrix element were then converted to polar coordinates (r, θ) by calculating radius $r = \sqrt{x^2 + z^2}$ and angle $\theta = \tan^{-1}(\frac{x}{z})$ and converting to degrees angle. A windrose for each plant root distribution (Supplemental Fig. S6) was generated by creating a histogram summing the total root pixels in the polar matrix in wedges of 45 degree increments (thus 8 canonical wedges were generated for each plant). A visualization of root distributions were obtained through production of windrose diagrams using R (programming language) [8].

References

1. Canny, J. A computational approach to edge detection. *IEEE Trans. Pattern Anal. Mach. Intell.* **1986**, *8*, 679–698.
2. Doi, K. Computer-aided diagnosis in medical imaging: Historical review, current status and future potential. *Comput. Med. Imaging Graph. Off. J. Comput. Med. Imaging Soc.* **2007**, *31*, 198–211.
3. Forsyth, D.; Ponce, J. *Computer Vision*; Pearson Education Inc.: Upper Saddle River, NJ, USA, 2003.
4. Gonzalez, R.; Woods, R. *Digital Image Processing*, 3rd ed.; Pearson Education Inc.: Upper Saddle River, NJ, USA, 2008.
5. Meyer-Baese, A. *Pattern Recognition in Medical Imaging*; Academic Press, Inc.: Orlando, FL, USA, 2003.
6. Privitera, C.M.; Stark, L.W. Algorithms for defining visual regions-of-interest: Comparison with eye fixations. *IEEE Trans. Pattern Anal. Mach. Intell.* **2000**, *22*, 970–982.
7. R-Core-Team. *R: A Language and Environment for Statistical Computing, 3.4.0*; R Foundation for Statistical Computing: Vienna, Austria, 2017.
8. Schneider, C.A.; Rasband, W.S.; Eliceiri, K.W. Nih image to imagej: 25 years of image analysis. *Nat. Meth.* **2012**, *9*, 671–675.
